# Editors’ Book Table

**Published:** 1872-11

**Authors:** 


### Editors' Book Table.

[Note.—All works reviewed in the columns of the Chicago Medical Journal may be found in the extensive stock of W. B. Keen, Cooke & Co., whose catalogue of Medical Books will be sent to any address upon request.]

BOOKS RECEIVED.

*The Principles and Practice of Surgery.* By Frank Hastings Hamilton, A.M., M.D., LL.D., Professor of the Practice of Surgery, etc., in Bellevue Hospital Medical College; Visiting Surgeon to Bellevue Hospital, etc., etc. Illustrated with 467 engravings on wood. New York: William Wood & Co., 27 Great Jones Street. 1872. Pp. 943.

We anticipated that Prof. Hamilton's work on Surgery would be of such a character as to constitute, with his works on Fractures and Military Surgery, a complete system of Surgery. Instead of this, we have a work which was intended by the author "to supply, within the compass of a single volume of moderate size, the instruction necessary to a full understanding of all the subjects belonging properly and exclusively to Surgery: the volume being intended as a text-book for students, and, at the same time, as a direct and complete guide to the surgeon."

The work which Prof. Hamilton thus marked out for himself is one exceedingly difficult of accomplishment. To attempt at the present time, to put in a "single volume of moderate size" *all* which is "necessary to a full understanding" of Surgery, would require an ability to condense which is rarely vouchsafed to man. We are fully impressed with the idea that expurgation is extensively required and can be profitably employed in surgical literature, but hardly, we opine, to an extent sufficient to enable an author to include *all* which is "necessary to a full understanding of all the subjects belonging properly and exclusively to Surgery." A careful examination of the book before us, while exciting our admiration for the degree of success which the author has achieved, awakens a regret that his ambition lay in the *condensing* direction. We regret that the book was not intended as a companion to Prof. Hamilton's former works, instead of covering very imperfectly the ground occupied by them. As a text-book for students the book is valuable; as a guide to the practicing surgeon it is "direct" and safe (as Prof. H. usually is), but it is hardly "*complete*" in either character. It is highly creditable to the author and to American authorship; but it has not realized its conception, and its failure to do so is in consequence of the conception being impracticable. We repeat: the work is valuable; but not as valuable as it would have been had the author's aim been higher.

*A Manual of Histology.* By Prof. S. STRICKER, of Vienna, Austria, in co-operation with Th. Meynert, F. Von Recklinghausen, Max Schulze, W. Waldeyer, and others. Translated by Henry Power, of London; James J. Putnam and J. Orne Green, of Boston; Henry C. Eno, Thomas E. Satterthwaite, Edward C. Seguin, Lucius D. Bulkley, Edward L. Keyes, and Francis E. Delafield, of New York. American Translation edited by Albert H. Buck, Assistant Aural Surgeon to the New York Eye and Ear Infirmary. With 431 illustrations. New York: William Wood & Co., 27 Great Jones Street. 1872.

*A Hand-Book of Post-Mortem Examinations, and of Morbid Anatomy.* By FRANCIS DELAFIELD, M.D., Curator to Belleville Hospital, Pathologist to the Roosefelt Hospital, etc., etc. New York: William Wood & Co., 27 Great Jones Street. 1872.

*History of Medicine, from the Earliest Ages to the Commencement of the Nineteenth Century.* By ROBLEY DUNGLISON, M.D., LL.D., late Prof. of the Institutes of Medicine and Medical Jurisprudence in Jefferson Medical College, etc., etc. Arranged and edited by Richard J. Dunglison, M.D. Philadelphia: Lindsay & Blakiston. 1872.

*Practical Lessons in the Nature and Treatment of the Affections produced by the Contagious Diseases;* with an account of the Primary Syphilitic Poison, and of its Communicability, based on extensive, direct, and comparative observation of the diseases in both sexes. With an Appendix, on the recent Report of the Royal Commission on the Contagious Diseases Act, and its application to the Voluntary Hospital System. By JOHN MORGAN, A.M., M.D., University of Dublin; Fellow of the Royal College of Surgeons, Ireland; Surgeon to Mercers Hospital and to the Westmoreland Lock Hospital, Dublin, etc., etc. With plain and colored illustrations. Philadelphia: J. B. Lippincott & Co. London: Bailliere, Tindall & Cox. 1872.

*Lectures, Clinical and Didactic, on the Diseases of Women.* By R. LUDLAM, M.D., Prof. of Obstetrics, and the Diseases of Women and Children, in the Hahnemann Medical College of Chicago; late President of the American Institute of Homœopathy, etc., etc. Second edition. Chicago: C. S. Halsey. Philadelphia: F. E. Boericke. London: Homœopathic Pub. Co. 1872.

*Ovarian Tumors: their Pathology, Diagnosis, and Treatment, especially by Ovariotomy.* By E. RANDOLPH PEASLEE, M.D., LL.D., Prof. of Gynæcology in the Medical Department of Dartmouth College; Attending Surgeon of the New York State Woman's Hospital, etc., etc. With fifty-six illustrations on wood. New York: D. Appleton & Co., 549 and 551 Broadway. 1872.

*General and Differential Diagnosis of Ovarian Tumors, with Special Reference to the Operation of Ovariotomy; and Occasional Pathological and Therapeutical Considerations.* By WASHINGTON L. ATLEE, M.D. With thirty-nine illustrations. Philadelphia: J. B. Lippincott & Co. 1872.

*The Science and Practice of Medicine.* By W. AITKEN, M.D. Third American, from the Sixth London edition. With additions descriptive of certain Forms and Types of Disease peculiar to this Country, and their Modes of Treatment. Edited by Meredith Clymer, M.D. 2 vols. Illustrated. Royal 8vo. $12; leather, $14. Philadelphia: Lindsay & Blakiston. 1872.

*Epidemic Cerebro-Spinal Meningitis.* With an Appendix, on some points on the Causes of the Disease, as shown by the History of the Present Epidemic in the City of New York. By MEREDITH CLYMER, M.D., University of Pennsylvania; Fellow of the College of Physicians of Philadelphia; formerly Physician to the Philadelphia Hospital, etc., etc. Philadelphia: Lindsay & Blakiston. 1872.

PAMPHLETS.

*Facts of Vital Statistics in the United States; with Tables and Diagrams.* Extracts from an Address by J. M. TONER, M.D. Published in the Circular of Information of the United States Bureau of Education for March, 1872.

*Transactions of the Medical Society of the State of Pennsylvania*, at its Twenty-Third Annual Session, held at Franklin, Pennsylvania, June, 1872. Volume IX, Part I. Published by the Society. Philadelphia: Collins, Printer, 705 Jayne Street. 1872.

